# Improved Fecundity in Northern China: A Secular Trend from 1980 to 2003

**DOI:** 10.1371/journal.pone.0165097

**Published:** 2016-10-27

**Authors:** Xiaobing Tian, Jingmei Jiang, Jiedong Wang, Shucheng Zhang

**Affiliations:** 1 North Sichuan Medical College, Nanchong, Sichuan, China; 2 Department of Epidemiology and Statistics, Institute of Basic Medical Sciences of Chinese Academy of Medical Sciences, School of Basic Medicine of Peking Union Medical College, Beijing, China; 3 National Research Institute for Population and Family Planning, Beijing, China; TNO, NETHERLANDS

## Abstract

**Objective:**

This study aimed to assess the trend of human fecundity over time in China.

**Methods:**

This retrospective study was conducted in Tongliao, China. Couples who were married during the time period between January 1, 1981 and December 31, 2003 were considered eligible for this study. A total of 27,413 individuals provided valid information via house-to-house interviews. The 12-month cumulative pregnancy rate (CPR) and annual percentage change were used as the outcome measurements.

**Results:**

There was a significant increase in the CPR over the five successive time groups. A break point in 1988 divided the entire study period into two distinct segments: 1981–1988, during which the CPR increased from 72.2% to 84.2%, and 1988–2003, during which the CPR increased from 84.2% to 87.2%.

**Conclusions:**

The findings were unlikely to be the result of biases, and could not be explained by increased medical treatment for infertility and changes in the prevalence of sexually transmitted diseases. Dramatic societal and behavioral changes due to the unique family planning policy and economic reform policies in China might have been the plausible reason for the results.

## Introduction

Evidence of a secular decline in sperm concentrations in some populations [1–4], together with the growing demands for assisted reproductive technology (ART) [5–7], have led to the conjecture that human fecundity, defined as the probability of a couple conceiving within a menstrual cycle, has been declining in recent decades. However, the findings of population studies conducted in Europe and the USA were contrary to expectations, and indicated that human fecundity has either improved or remained unchanged over the last few decades [8–10]. Although heterogeneity in the methodologies [11] and spatial variation [12, 13] were plausible explanations, the actual extent and time trend of human fecundity remain unknown.

Time to pregnancy (TTP), which is defined as the number of months or cycles of unprotected intercourse that a couple take to conceive [11], has been highly accepted and extensively used in epidemiological studies to assess the impact of volitional factors on fecundity and to examine the time trend of fecundity [14, 15]. Based on retrospective reports, information on the TTP was easy and inexpensive to obtain via questionnaires and interviews [16]. However, the accurate measurement of the TTP requires subjects to recall the beginning and end of the time period explicitly [11]. As some pregnancies are the result of contraceptive failures or unprotected sex without a clear pregnancy plan, TTP data in these cases cannot be collected with equivalent quality as the data from individuals who have planned their pregnancy [17]. Furthermore, there is a high possibility that unplanned pregnancies occur among individuals with a high fecundity [16]. Therefore, restricting the analyses to planners would result in a selection bias due to over-sampling of individuals with sub-fecundity. Additionally, some couples might recall or report incorrectly by mentioning unplanned pregnancies as planned, resulting in incorrect measurement of the TTP [16]. Moreover, couples not intending to conceive had a lower degree of protection in earlier years than their later counterparts due to the lower availability and poorer quality of contraceptive methods [18, 19]. The proportion of women who decide to terminate unintended pregnancies has also altered over time because of societal changes and consequent changes in reproductive attitudes [20]. Therefore, this type of bias could not be eliminated solely using a standard study design.

In China, the world’s most stringent family planning policy and program for slowing down birth rates, which limited most couples to having one child, was implemented in 1980. Under this so-called one-child policy, unplanned pregnancy was rare and unintended birth was strongly discouraged. Therefore, TTP data based on this population would be subject to less planning-related bias. During the same period, another cornerstone national policy termed the “Economic Reform Policy,” which stipulated that the emphasis should be shifted toward economic development and encouraged people to pay more attention to things beyond politics, was established and brought about dramatic socioeconomic changes. We hypothesized that these changes might have potential influences on fecundity. Therefore, the present cross-sectional study aimed to examine whether there was a secular trend in fecundity among female residents of Tongliao, China in the period of 1981–2003.

## Methods

### Study population

This survey was conducted from December 2008 to May 2011 in Tongliao, an administrative district of the Inner Mongolia Autonomous Region of China with an approximate population of 3 million, which is unequally distributed among its nine affiliated counties. Tongliao has a similar population structure, climate and environmental conditions, economic structure, medical policies, and lifestyle to general rural areas in northern China.

**Fig 1:**
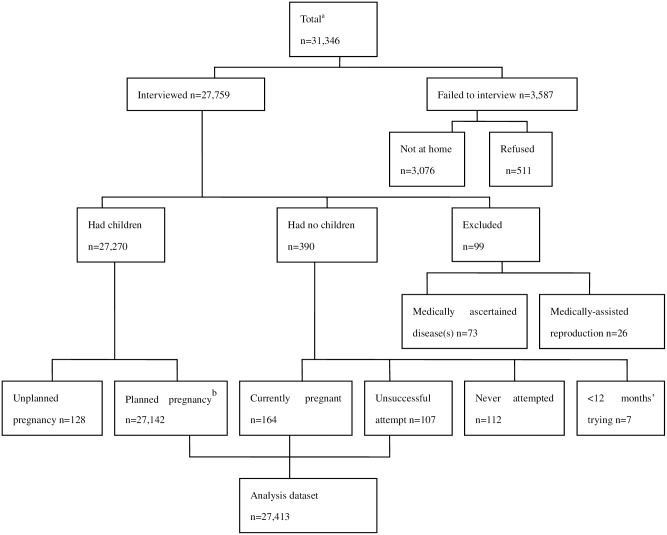
Selection process for subjects with eligible time to pregnancies. ^a^Data were collected from marriage registers in the sampled towns. ^b^Included those who became pregnant after >12 months of attempting to conceive.

The inclusion criteria for this study were as follows: individuals residing in local rural communities or villages; individuals who were married during the period between January 1, 1981 and December 31, 2003 (including legal marriage and facto marriage); and individuals who had started attempting to conceive during the same period. Individuals who had medically ascertained reproductive abnormities, such as endometriosis and anovulation, as well as those who had undergone an ovariectomy or a hysterectomy, were excluded from the study (Fig 1). The minimum sample size for each year (1981–2003) was predetermined as 800, as previous studies [10, 16, 21] suggested that this was sufficient to reach the desired statistical power. The sample was proportionally derived from each county according to the number of eligible women in that county based on the county’s marriage register. Within each county, two-stage cluster sampling was performed to select study sites, using rural towns and villages as sampling units. The number of sample units was determined based on our pilot study, with the consideration that the total number of subjects in the final sample would not be lower than the predetermined year-specific sample size.

### Study questionnaire

We collected the required information for our study retrospectively using an interviewer-administered questionnaire (See S1 and S2 Files), which was piloted in three rural communities other than the selected sites. The questionnaire sought information on age, age at pregnancy, occupation, ethnicity, education, smoking history, and parity. For the collection of detailed information on the TTP and pregnancy outcomes among those who had children or who were pregnant, we focused on the first pregnancy and asked the following question: “How many months or years did it take you to become pregnant?” This question was preceded by filter questions regarding oral contraceptive use and was followed by a question regarding the outcome of the pregnancy (abortion, stillbirth, or living birth). Alternatively, childless individuals were asked: “Had you ever tried to conceive for >12 months”. Those who answered ‘yes’ on this question were defined as involuntarily childless and were included in the analysis, while those who responded negatively to this question were categorized as voluntarily childless and were excluded. TTP values were eligible for inclusion if conception was not the result of contraceptive failure (Fig 1).

### Data collection

This study recruited technical employees from village-level family planning service stations who were high school graduate or above, had completed at least three valid pilot questionnaires, and had received additional training to conduct interviews in their own village for accurate acquisition of information. These people were selected as interviewers in order to maximize the response rate, as they were familiar with the social and cultural backgrounds, the local subjects, and the underlying aspects, since their routine work involved providing reproduction-related services, including birth registration and management of the study population. Interviewers visited each house in their community and conducted face-to-face interviews with eligible women. Interviewers also completed the questionnaire on behalf of illiterates as per the verbal interview. Quality control procedures were followed throughout the study by daily checks of the quality and completeness of the study group. A second interview was conducted for correction of invalid/incomplete questionnaires.

After a detailed description of the study procedures, written informed consent was obtained from each participant. The study content and procedures were reviewed and approved by the Human Research Ethics Committee of Peking Union Medical College.

### Statistical analysis

We first categorized the time duration from the year at the start of the attempt to conceive into five subgroups with 5-year intervals: 1981–1985, 1986–1990, 1991–1995, 1996–2000, and 2001–2003. The final group covered a period of 3 years and included women who started attempting to conceive after 2003 because of the small proportion of these individuals. The maternal age at the start of the attempt to conceive was also categorized into three subgroups: ≤19 years, 20−25 years, and ≥26 years. Stillbirths and spontaneous abortions were grouped as adverse outcomes of pregnancy. Ethnicities other than Han and Mongolian were combined, as there were too few women in other racial groups in Tongliao. For the same reason, education levels above high school were also combined.

Next, we analyzed the TTP via survival analysis techniques using the Kaplan-Meier method, stratified by time groups. The cumulative pregnancy rates (CPRs) were then computed from these estimates. The TTP was censored after 12 months, which was the time when infertility treatment was normally initiated. The log-rank test was performed to examine the differences in CPRs among the time groups.

Trends in the TTP were analyzed by considering the time groups as regression variables in Cox’s proportional hazards model for a discrete time scale. This process generated odds ratios (ORs) for which values >1 indicated a higher fecundity. The unadjusted hazard ratios were estimated using the first group (1981–1985) as a reference, after which multivariate analyses were performed with adjustment for the effect of the following potential confounding factors: maternal and paternal age at the start of the attempt to conceive, female education level, occupation, maternal smoking habit, and ethnicity. Backward selection was used to exclude variables without significant effects at *α* = 0.20. As a result, maternal smoking and ethnicity were kept in the final model. The use of oral contraception was not included in the final model, since the contraceptive pill tends to decrease fecundity in the early months after discontinuation of use [22], violating the assumption of proportional hazards.

We implemented the Joinpoint regression model [23] to describe changes in fecundity over time, with the consideration that human fecundity might not decrease or increase smoothly over a long period of time.

A descriptive analysis and Cox’s proportional hazard model were performed in SAS 9.1 (SAS Institute Inc.; Cary, NC, USA: 2004). The Joinpoint regression model was performed in the Joinpoint Regression Program, version 3.4.3 (National Cancer Institute, 2010).

## Results

A total of 31,346 women were deemed suitable from the local marriage registers. Among these, 27,759 women participated in the survey and 3,587 failed to be interviewed (Fig 1); the response rate was 88.6%. Of the eligible participants, 128 women who reported contraceptive failure were excluded, and 112 individuals who claimed to have no intention of having a child were also omitted. Also excluded from the analysis were 7 responders who were planning a pregnancy at the time of the survey. Finally, 27,413 subjects were included in the main data analysis, of which 73.2% attempted to conceive in the first month after marriage and 26.8% used contraception prior to the attempt. The median duration of contraceptive use was 10 months (range, 1–96 months).

The proportion of women who were aged >26 years when first attempting to conceive increased over time, while those who were aged ≤19 years decreased over time. A decline in the percentage of women who had an education level below junior school and a rise in those who had junior school education were also observed. The composition of occupations, as well as the proportions of subjects using oral contraceptives, remained unchanged over time. Furthermore, the percentage of Han women decreased, while that of Mongolian women increased, over time. The occurrence of adverse outcomes in the first pregnancy remained at a low level, but exhibited an increasing trend over time (Table 1).

**Table 1:** Percentage distributions of factors affecting fecundity among married women in Tongliao, China over time.

Factor	Time group					P-value
	1981–1985	1986–1990	1991–1995	1996–2000	2001–2003	
	(n = 4689)	(n = 5268)	(n = 6548)	(n = 6389)	(n = 4519)	
Female age at the start of the attempt (years)						<0.01
≤19	11.60	5.88	3.39	2.95	1.10	
20–25	87.50	86.85	85.96	84.98	86.60	
≥26	0.89	7.27	10.65	12.07	13.39	
Education level						<0.01
Primary school or below	25.47	23.92	22.64	19.64	15.29	
Junior school	64.11	67.24	68.02	71.39	75.95	
High school or above	10.41	8.85	9.34	8.97	8.76	
Use of OC before attempt	2.19	1.90	1.36	2.04	1.50	0.21
Occupation						0.24
Manual labor	88.72	87.24	87.71	87.28	86.92	
Mental labor	11.28	12.76	12.29	12.72	13.08	
Maternal smoking	2.20	1.75	1.10	1.03	1.62	<0.01
Ethnicity	2.10	3.50	3.10	2.60	2.70	<0.01
Han	58.91	57.67	55.92	52.36	49.72	
Mongolian	37.71	40.02	41.56	44.93	46.89	
Other	3.38	2.32	2.52	2.71	3.39	
Adverse outcomes of first pregnancy	1.61	2.97	3.56	3.65	4.29	<0.01

OC, oral contraceptive.

The CPR was observed to be increasing over time, and this trend could be seen for each month among the year groups (each row of Table 2). The CPRs of individuals with sub-fecundity (TTP>12 months) were 23.23%, 17.56%, 14.43%, 13.06%, and 12.97% for the 1981–1985, 1986–1990, 1991–1995, 1996–2000, and 2001–2003 time groups, respectively, displaying an evident decline over time (Table 2). The increasing trend in fecundity was further confirmed by calculating ORs, which were >1 for each year group (with reference to the first year group, 1981–1985) and were increased over time. After adjusting for potential confounders like maternal smoking and ethnicity, this upward trend were slightly strengthened (Table 3).

**Table 2:** CPRs over 12 months among married women in Tongliao, China, according to the time group.

	CPRs for time groups					
Month	1981–1985	1986–1990	1991–1995	1996–2000	2001–2003	Total
	(n = 4689)	(n = 5268)	(n = 6548)	(n = 6389)	(n = 4519)	(n = 24713)
1	29.12	32.50	36.55	36.78	38.55	35.44
2	43.44	47.42	52.13	53.47	53.73	51.00
3	52.4	57.02	61.39	62.67	63.44	60.35
4	58.16	62.81	66.43	68.17	68.33	65.71
5	61.88	67.08	70.75	72.42	72.91	69.98
6	65.53	70.33	74.21	76.11	77.38	73.65
7	67.76	73.12	76.71	78.55	80.15	76.20
8	69.77	75.4	78.68	80.77	82.01	78.29
9	71.48	77.26	80.64	82.47	83.65	80.08
10	74.15	79.52	82.90	84.65	85.09	82.22
11	75.9	81.32	84.57	85.94	86.24	83.74
12	76.87	82.44	85.57	86.94	87.03	84.73

Log-rank test: *x*^*2*^ = 222.08, P<0.01. CPR, cumulative pregnancy rate.

**Table 3:** Crude and adjusted odds ratios (95% CIs) for time to pregnancies in relation to the first time group.

Time group	Odds Ratio (95% CI)	
	Unadjusted	Adjusted^a^
1981–1985	1	1
1986–1990	1.19 (1.12−1.26)	1.19 (1.12−1.26)
1991–1995	1.32 (1.25−1.40)	1.33 (1.25−1.40)
1996–2000	1.39 (1.32−1.47)	1.40 (1.32−1.48)
2001–2003	1.40 (1.31−1.49)	1.41 (1.32−1.50)

^a^Maternal smoking (yes/no) and ethnicity (Han/Mongolian/other) were kept in the final regression model after stepwise selection. Maternal smoking (reference = no): OR = 0.89 (95 CI: 0.86−0.92). Maternal ethnicity (reference = Han): Mongolian OR = 0.92 (95% CI: 0.90−0.95); other OR = 0.94 (0.86−1.03). CI, confidence interval.

**Fig 2:**
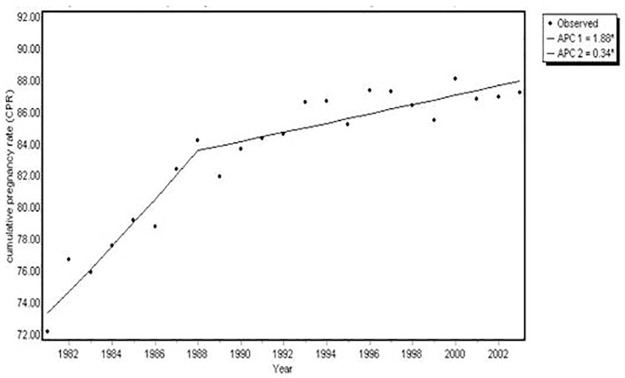
Trend of fecundity among married women, 1981–2003, Tongliao. APC, annual percentage change.

The fecundity rates from 1981−2003 are presented in Fig 2. A break point was observed in 1988, which split the entire study population into two segments of time: 1981–1988 and 1988–2003. Fecundity significantly increased during the first segment from 72.2% to 84.2%, followed by a slight increase in the second segment from 84.2% to 87.2%. The annual percentage changes were 1.88% and 0.34% during 1981–1988 and 1988–2003, respectively.

## Discussion

The present study found a clear increasing trend for fecundity from 1981−2003 among female residents of Tongliao, China using retrospectively collected TTP information. The most interesting finding in this study was that the fecundity was found to be increasing in a non-linear manner; a break point in 1988 divided the entire period into two distinct segments, comprising a rapidly increasing period followed by a less rapidly increasing period. However, the information collated in this study could not sufficiently explain this trend.

In a previous study, truncation bias was shown to have the potential of suggesting an artificial increase in fecundity when it in fact remained unchanged or was decreased [16]. This bias resulted from systematically including less fertile couples in the early years of the study period and/or excluding them from the study population in recent years [18]. In the present study, the ascertainment of couples who were having unprotected intercourse was defined as the ‘starting time’, rather than depending on the occurrence of conception or birth, thus making left truncation bias unlikely. Similarly, we predetermined a 5-year gap between the upper limit of the study period (December 2003) and the investigation time (December 2008) in order to minimize potential right truncation bias. This gap was sufficient to observe the outcomes of attempts from the majority of eligible couples. Therefore, truncation bias was not a feasible explanation for the result.

Protection bias was another possible explanation for this rising trend; fewer fertile couples were excluded from TTP distributions in later years than their earlier counterparts due to an increased ability to prevent unintended births over time [19]. We considered that this bias would have limited influence on the results of the present study, although we could not determine the actual time trend of elective abortions, mainly as a result of the incomplete medical records of induced abortions from local medical institutes and unreliable reports from participants. During the earlier years of the study period, premarital sex was not culturally accepted in China; those who had been pregnant or given birth prior to marriage were severely stigmatized. Furthermore, couples not intending to have a child had easy access to free contraception services provided by family planning centers. In addition, unlike some western countries where induced abortions were not legally permitted, termination of unauthorized pregnancies has long been encouraged in China and the relevant services are provided by many family planning clinics [24]. In the present study, only 128 responders reported accidental pregnancy, providing further evidence for this.

As a retrospective study, it was necessary to consider the potential for having recall bias. Validation studies suggested that the responders had given an accurate representation of the true TTP distribution, even with recall of up to 20 years [25, 26]. This was also practically plausible since the first pregnancy and birth were naturally a very important event for every couple. This was especially the case in China, where the one-child policy meant that one couple could only have one child. In addition, no studies on fecundity have reported that recall bias might lead to differential errors. Therefore, it is unlikely that recall bias was an explanation for this trend, although, as the longest recall was 28 years, it could not be ruled out.

In the present study, responders were not asked whether their pregnancy was the result of medical assistance. This raises the concern that the TTP distribution might demonstrate less fecundity, since successful medical assistance would have added additional pregnancies to the data and such pregnancies tend to have a longer TTP [16]. However, we speculated that it would not have a considerable influence on the time trend of fecundity. As the rapid development in ART occurred only during the last decade in China [27], it has not been culturally and financially accepted by Chinese couples, especially for those living in rural areas [28]. Additionally, we censored couples who had a TTP of >12 months, a duration within which couples were unlikely to seek medical help for infertility [29]. Moreover, if medical treatment did influence the results, it would have underestimated the trend over time.

We found that changes in demographic characteristics, including ethnic distribution, the average age at first marriage, and the rate of oral contraceptive use, were approximately identical to the population characteristics from statistical reports or specific surveys of the area [30]. Therefore, we considered the study sample to be a good representation of the underlying population. Some factors, including maternal age at first attempt and education level, had no significant influence on fecundity, which seemed inconsistent with previous studies [8, 9]. This might be due to the fact that the overall age at attempt was within the fertile age range, and the overall variation in the education level was too low to be a critical determinant of occupation and economic status. The changes in demographic characteristics might in part, but not completely, explain this trend. Although some factors favoring fecundity, including education level, displayed an increasing trend, and some unfavorable factors, such as the prevalence of maternal smoking and oral contraceptive use, were decreasing or remained unchanged, the magnitude of changes for each factor was too small to significantly impact on the fecundity trend.

Some researches attributed the increase in fecundity to the decline in the prevalence of STDs, such as gonorrhea [18] and chlamydia [9]. However, the incidence of STDs has increased significantly and demonstrated a nonlinear growth trend in China. For example, the incidence of gonorrhea increased from 9.49 in 1989 to 23.97 in 1998 per 100 000 people [31]. During the same period, the incidence of syphilis increased 20-times, from 0.17 to 4.31 per 100 000 people. Nongonococcal urethritis/cervicitis also increased from 0.51 to 9.74 per 100 000 people [32]. Although the Chinese public health reporting system did not track chlamydia infections, its increasing trend could be deduced from some regional studies [33]. Moreover, the economic and socio-cultural environment led to a rapid out-migration of rural labor and rapid changes in sexual beliefs and behaviors in young persons, which might facilitate the transmission of STDs. Therefore, the increase in STDs might be a plausible explanation for the slowing upward trend of fecundity after 1988.

Given the dramatic societal and behavioral changes during this period, we anticipated that these changes might also have some favorable effects on fecundity, which could be used to explain the rising trend. With the implementation of the one-child policy in 1980, two major medical service systems, including the family planning system and the general health system, were gradually established at the grass-roots level [34]. These systems were responsible for public education on contraceptive use and reproductive health, the distribution of contraceptive measures, and the training of grass root level family planning providers. With the enhanced knowledge of factors such as ovulation and timing of intercourse gained from this program, couples might have learnt how to plan their pregnancy in a healthier and more efficient manner, and how to prevent accidental pregnancy to a greater extent. Moreover, as the majority of couples could only have one child, they would likely have put greater effort into improving the quality of birth through active adjustment of behaviors, such as quitting smoking and alcohol. In addition, environmental and living conditions were considerably improved in Tongliao during the period of study. According to the statistical bureau of Tongliao [35], the gross domestic product per person and the household disposable income increased from 369 Yuan (~62 USD) and 105 Yuan (~15 USD), respectively, in 1980 to 25,402 Yuan (~4,097 USD) and 2,779 Yuan (448 USD) in 2003, respectively. Meanwhile, the population size increased from 2.36 million in 1980 to 3.09 million in 2003. In 2003, >37% of rural residents engaged in non-agricultural employment, which was significantly higher compared with 15.8% in 1980. **T**he remarkable changes in recent decades might have also promoted the health status of individuals and strengthened the capability of the public to use medical services, which consequently was favorable for reproduction.

Notably, the probability of becoming pregnant in a menstrual cycle, which was defined as fecundity in the present study, is also dependent on other volitional factors than the couple’s biological fecundity, including the timing and frequency of unprotected intercourse. However, as a retrospective study, we could not collect information on these factors. The absence of adjustment for these confounders might undermine the comparability of this study with studies from populations with different cultures and from different regions, particularly in the case of any systematic shift in the levels of these factors. Prospective studies offer advantages with respect to the collection of data on volitional factors, as well as the accurate timing and assessment of exposure in relation to events during the menstrual cycle, fertilization, and implantation. However, prospective studies carry the burdens of high costs, which are unfeasible in under-resourced settings, such as China. Moreover, we did not assess the influence of pre-marital sex and elective abortion on the trend of fecundity, mainly due to incomplete medical records and the incredible of the information provided by participants. Further studies are needed to clarify this.

## Conclusion

In conclusion, we found an increasing trend of fecundity in a regional population in northern China during 1981–2003. This upward trend was unlikely the result of biases. Societal and behavioral changes as a result of family planning and economic reform policies might have been the probable explanations.

## Supporting Information

S1 File Questionnaire Chinese version. (DOC)

S2 File Questionnaire English version. (DOC)
