# Supplementary material for: Improved Fecundity in Northern China: A Secular Trend from 1980 to 2003
Source: PLoS One. 2016 Oct 27;11(10):e0165097. doi: 10.1371/journal.pone.0165097 (PMC5082934; doi:10.1371/journal.pone.0165097)
Supplement: S1 File — (DOC) [file pone.0165097.s001.doc]

**已婚人群生育力调查问卷**

**(仅供已婚妇女使用)**

非常感谢您能参加我们的调查。这次调查是育龄妇女生育力研究项目的一个部分，我们拟以此评估人群生育力现状以及相关影响因素。为了保护你的隐私，只有经过培训并经研究组指定的人才能对您进行调查，而且，您提供的所有信息将严格保密，且只用于本次研究数据分析。

这次研究是由**国家人口与计生研究所**负责实施，负责人为**张树成**教授，如果您有不清楚或想进一步了解相关信息，可以跟他联系：

电话：010-62170085 电子邮件：[nrifp1@263.net](mailto:nrifp1@263.net)

**问卷编号**______

**姓名** __________ **居住地** ________(县、区、旗)____镇_____村

**调查员**____________ 调查日期_____月_____日

**一、基本情况**

1. 您的出生年月 _______年_______月 (例：1982年5月)
2. 您的民族

1）汉族 2）蒙古族 3) 满族 4)朝鲜族 5) 达斡尔族 6)其他

1. 您的文化程度

1）小学及以下 2）初中 3）高中（中专） 4）大专及以上

1. 您现在的职业

1）农民 2）工厂工人 3）工厂（行政）管理人员 4）机关干部 5）教师 6）商业服务业 7）公司职员 8）其它____________

1. 您的结婚日期__________年________月 (例：***1982***年***5***月)
2. 你是否有经医生确认的影响生育的疾病？

1）有 2）无

1. 你是否有孩子？

1）有 ______个 2）无

**二、计划怀孕情况**

**计划怀孕是指夫妻双方以生育为目的，有规律性行为而且停止任何避孕措施。**

1. 结婚后，您是否有过怀孕计划？

1）有 2）无

**回答无，跳至第三部分。**

**下列问题是关于你计划怀孕的相关情况，如果您有多次怀孕计划，以下是第一次计划怀孕时的情况。**

1. 计划怀孕时，您的年龄是____(岁)
2. 计划怀孕期间，您是否吸烟？

1）是 2）否

1. 计划怀孕期间，您是否经常饮酒？

1）是 2）否

1. 计划怀孕之前，您是否采用避孕措施？

1）使用 2）未使用

11.1 如使用，您**最后**使用的避孕措施是

1）避孕套 2）口服避孕药 3）节育环 4）安全期避孕 5）其它

11.2 计划怀孕前，您共避孕________月？

1. 您这次怀孕计划的结果是
   1. 怀孕 2）未怀孕

**(回答2， 跳至问题15)**

1. **如怀孕成功**，从计划怀孕（停止避孕）开始到确认怀孕，共经历了多长时间？

共 _______年_________月

怀孕计划开始时间________(年)_______(月) *（如：****1982****年****5****月）*

确认怀孕时间 ________(年)_______(月) *（如：****1982****年****10****月）*

1. 如果**未能**怀孕成功，从怀孕计划（停止避孕）开始，你共经历了多长时间？

1）<6 月 2）6-12月 3）12-24月 4）24月以上

1. 计划怀孕期间，你是否使用过医学辅助怀孕措施？

1）是 2）否

1. 如果怀孕成功，这次的怀孕结局是：

1）正在怀孕期间 2）足月生产 3）早产 4）自然流产 5）人工流产 6）死胎 7）其它_____

1. 这次怀孕的生育情况是：

18.1 孩子出生日期_____(年)______（月） （如：**1982**年**5**月）

18.2 孩子性别 1）男 2）女 3）双胞胎

18.3 出生体重__________克，身长________厘米

18.4 出生后有无确诊的异常？

1）无 2）有 __________________________________

**三、无怀孕计划的原因**

1. 你从未有过怀孕计划，其原因是

1) 因病无法生育 2）不打算要孩子 3）延迟要小孩（晚育） 4）其他

1. 您是否怀过孕？

1）是 2）否

**调查结束，谢谢您的配合。**
