# Supplementary material for: Improved Fecundity in Northern China: A Secular Trend from 1980 to 2003
Source: PLoS One. 2016 Oct 27;11(10):e0165097. doi: 10.1371/journal.pone.0165097 (PMC5082934; doi:10.1371/journal.pone.0165097)
Supplement: S2 File — (DOC) [file pone.0165097.s002.doc]

**Female fecundity Questionnaire**

**(For Married Women)**

Thank you for your participation in this study! We appreciate your willingness to participate in this important research. This questionnaire is part of a study to assess the fecundity of population. We are aiming to have a better understanding of human fecundity and explore the potential factors impacting on the fecundity. To ensure your privacy, only authorized study personnel will have access to your responses. All answers are completely confidential and only used for research purpose.

This study is being conducted through the National Research Institute of Population and Family Planning. The Principal Investigator is Prof. Zhang Shucheng. He can be reached at 010-62170085 or [nrifp1@263.net](mailto:nrifp1@263.net). If you have questions regarding your rights as a research participant, or if problems arise which you do not feel you can discuss with the Investigator, you can contact with him.

Study ID#________

Name__________

Address________(county/district) ____Town _____Village

Interviewer_______(Signature) Date_____(MM)____(Day)

**A. General information**

1. Your birth date _____(MM)_______(YYYY)
2. Your ethnicity

(1) Han (2) Mongolian (3) Manchu (4) Korean (5) Daur (6) other____(Specify)

1. Your educational level

(1)  Primary school or below (2) junior school (3) high school/technical school (4) College or above

1. Your current occupation ________________
2. The date of your marriage _____(MM) ___(YYYY)
3. Do you have any medically ascertained diseases which lead to infertility?
4. Yes (2) No
5. Do you have child(ren)?
6. Yes , I have _____child(ren) (2) No

**B. Information of the pregnancy plan**

**Planed Pregnancy** means that you were attempting to be pregnant and having sexual intercourse with a male partner without using any method to prevent pregnancy.

1. Have you ever planned to get pregnant after your marriage?

(1) Yes (2) No

**If No, skip to part C.**

**The next set of questions will ask about your first time of “attempt” to conceive, whether or not this attempt actually resulted in a pregnancy.**

1. At the start of the pregnancy plan, your age? ______(years)
2. At the start of the pregnancy plan, were you a regular smoker?

(1) Yes (2) No

1. At the start of the plan, were you frequently drinking alcohol?

(1) Yes (2) No

1. Prior to pregnancy plan, did you use any kind of method to prevent pregnancy?

(1) Yes (2) No

12.1 If yes, what was the **last** type of birth control you used?

(1) condom (2) oral contraceptive pill (3) contraceptive ring (4) safe periodrhythm (5) others

12.2 How long did you use this method? ________year(s) ______Month(s)

1. Did this attempt result in a pregnancy?

(1) Yes (2) No

**(If No, skip to question 14)**

1. If yes, how many months or years did it take you to become pregnant?

______ (Years) _____(Months)

The start time of this attempt ______(MM) ______(YYYY)

Confirmation date of pregnancy ______(MM) ______(YYYY)

1. **If No,** how many months or years has it been since you initiated your pregnancy plan?

(1)<6 months (2) 6-12 months (3) 13-24months (4) >24 months

1. During the period of trying to conceive, did you use any kind of assisted reproductive technology?

(1) Yes (2) No

1. If you succeeded in pregnancy, what is the outcome of this pregnancy

(1)Currently pregnancy (2)term birth (3) preterm birth (4) spontaneous miscarriage (5)induced abortion (6) stillbirth (7) others

1. The following is about the reproductive outcome for this pregnancy

18.1 Date of birth: _____(YYYY)_______(MM)

18.2 Birth weight:________(g)

18.3 Birth height: ________(cm)

18.4 Gender: (1) male (2)female (3) twins

18.5 Abnormal birth outcome(s): (1)Yes (2)No

**C. Reasons for unwillingness to conceive**

1. What is the reason for your unwillingness to conceive?

(1) Incapable of pregnancy (2) unwillingness to have a child (3) postponement of childbearing (4) other reasons

20. Have you ever got pregnant?

(1) Yes (2) No

**(This is the end for this interview, thank you for your cooperation)**
